# Metabolic and Genetic Properties of *Petriella setifera* Precultured on Waste

**DOI:** 10.3389/fmicb.2018.00115

**Published:** 2018-02-08

**Authors:** Karolina Oszust, Jacek Panek, Giorgia Pertile, Anna Siczek, Marta Oleszek, Magdalena Frąc

**Affiliations:** Laboratory of Molecular and Environmental Microbiology, Department of Soil and Plant System, Institute of Agrophysics, Polish Academy of Sciences, Lublin, Poland

**Keywords:** *Petriella setifera*, genetic and catabolic diversity, lignocellulose utilization, waste debris

## Abstract

Although fungi that belong to *Petriella* genus are considered to be favorable agents in the process of microbial decomposition or as plant endophytes, they may simultaneously become plant pests. Hence, nutrition factors are supposed to play an important role. Therefore, it was hypothesized that *Petriella setifera* compost isolates, precultured on three different waste-based media containing oak sawdust, beet pulp (BP) and wheat bran (WB) will subsequently reveal different metabolic properties and shifts in genetic fingerprinting. In fact, the aim was to measure the influence of selected waste on the properties of *P. setifera.* The metabolic potential was evaluated by the ability of five *P. setifera* strains to decompose oak sawdust, BP and WB following the MT2 plate^®^ method and the catabolic abilities of the fungus to utilize the carbon compounds located on filamentous fungi (FF) plates^®^. Genetic diversity was evaluated using Amplified Fragment Length Polymorphism analysis performed both on DNA sequences and on transcript-derived fragments. *P. setifera* isolates were found to be more suitable for decomposing waste materials rich in protein, N, P, K and easily accessible sugars (as found in WB and BP), than those rich in lignocellulose (oak sawdust). Surprisingly, among the different waste media, lignocellulose-rich sawdust-based culture chiefly triggered changes in the metabolic and genetic features of *P. setifera*. Most particularly, it contributed to improvements in the ability of the fungus to utilize waste-substrates in MT2 plate^®^ and two times increase the ability to catabolize carbon compounds located in FF plates^®^. Expressive metabolic properties resulting from being grown in sawdust-based substrate were in accordance with differing genotype profiles but not transcriptome. Intraspecific differences among *P. setifera* isolates are described.

## Introduction

Fungi represent a large share of the total biodiversity on Earth and they are regarded as key players in performing numerous ecosystem functions, especially in soil. It has been established that there is a link between the soil fungal community structure and function and to extent to which the soil is intensively managed (Knoblauch et al., 2017). It is also widely acknowledged that organic waste, applied as biofertilizers or exogenous organic matter (EOM) is superior to chemical fertilizers at improving the biological quality of soil (Li R. et al., 2017). A wide range of biofertilizers have been proposed over the last couple of decades. These were, for example, mainly manure (Chen and Jiang, 2014), composts or biochar (Liu et al., 2012), waste from biogas plants (Minale and Worku, 2014), municipal sewage sludge (Tontti et al., 2017), dairy sewage sludge (Frąc et al., 2012), algae or zeolite (Türkmen and Kütük, 2017), bone meal (Chen et al., 2011), agricultural or food wastes (Zhang et al., 2011). It has been described previously that the microbial community structure of soil after long-term organic fertilization reveals important associations between nutrients and specific taxa involved in nutrient transformation which occurs in soil (Li F. et al., 2017). Land application is the best recycling option since most organic wastes contain valuable nutrients and organic matter which may be used to improve soil fertility.

However, adding organic waste to soil is not only useful due to the organic matter and nutrients introduced; also additional fungal species may be included with biofertilizers (Frąc et al., 2014). Whenever the indigenous microbial fungal species of the organic waste meet the appropriate conditions for growth and development, they may have pivotal ecological roles influencing plant health as symbionts or decomposers. On the other hand they may act as plant pathogens (Frąc et al., 2014), produce toxins, or even cause animal or human mycoses (Pascual et al., 2008). To the best of our knowledge the mycological compositions of biofertilizers are rather poorly described compared to the soil after its incorporation. However, biofertilizers may influence soil fungal biodiversity. Biofertilizers are more often analyzed to discover their physicochemical properties. This is primarily to prove their expected positive impact on soil or to evaluate the potential risks associated with pollution from heavy metals or toxic organic compounds. If the microbial composition is being taken into consideration, then the waste is characterized by the presence of pathogenic bacteria, viruses and parasites (Pascual et al., 2008), but very rarely fungi. In order to prevent the spread of pathogenic microorganisms, EU Directive 86/278/EEC asserted that some biofertilizers, e.g., sludge should be appropriately treated to satisfy specific microbial standards before it is applied to land. The EU has also specified use restrictions according to the type of treatment applied.

However, as the ascospores produced by fungi, belonging to Ascomycota are resistant to many chemicals and physical treatment, they can survive sanitization or disinfection applied prior to agricultural disposal and in consequence they can develop in soil (Frąc et al., 2015). Therefore, it seems reasonable to make the mycological characterization of biofertilizers more widespread and to reinforce restrictions in order to diminish the risks to human health, farm animals and wildlife from contaminated plants. It is not only important to name the taxa, but also to describe in detail the metabolic properties of ecotypes of genera inhabiting diverse biofertilizers.

This may be useful since more attention has been paid recently to the possibility of using filamentous fungi (FF) in the degradation and detoxification of waste incorporated into the soil (Mannan et al., 2005; Rahman et al., 2016). The great diversity of fungi occurring in soil and in various types of waste may be the source of isolates with diverse biotechnological properties, used in new technologies and in the acquisition of natural products (e.g., enzymes). To speed up the process of waste degradation and reduce the risk associated with the presence of potentially pathogenic fungi in these wastes it is possible to use selected strains of microorganisms to facilitate waste-based biomass decomposition, especially when it is applied onto the soil. It is therefore essential to provide research concerning the characterization of fungal strains isolated from waste (Frąc, 2012).

The species of *Petriella setifera* (Alf. Schmidt) Curzi, belonging to the Ascomycota phylum, Microascaceae family, is often found in the soil enriched with manure or composts (Danon et al., 2010; Lackner et al., 2014) this is interesting in two contexts: waste degradation and pathogenesis, however, to date, it has been rather poorly described. *P. setifera* is classified as a soft-rot-causing fungi. These are known to secrete cellulase from their hyphae, this is an enzyme that breaks down cellulose and hemicellulose in wood (Janusz et al., 2013), however, as was demonstrated previously, *P. setifera* also demonstrates the ability to degrade lignin, and therefore it may be regarded as a brown rot fungi (Mathieu et al., 2013). Although these fungi are important ecological agents in the process of nutrient recycling by microbial decomposition, they are classified as pests in their role as destructive agents of wood rot. The genus of *Petriella* was also described as a pathogen of oak twigs sessile and the bark of Scots pine (Kwaśna et al., 2005), and also a root endophyte of *Salvia miltiorrhiza* (Lou et al., 2013). Fungi do live as parasites, but if the plant dies, whether as a consequence of the fungal infection or not, the fungus continues to degrade the biomass without further need for parasitic activity (Agrios, 2005). *Petriella*, being a facultative parasite, usually conforms to this saprophytic activity but may resort to parasitic action, but does not absolutely rely on any host for the completion of its life cycle.

Finding *P. setifera* strains in industrial compost and knowing the fact that this genus is simultaneously a saprophyte and a parasite, we assumed that *P. setifera* may have a strong tendency to live or to degrade not only oak but also wheat or sugar beet. The fact is the environmental factors, among others the nutritional constraints, alter the catabolic and genetic properties of microorganisms. These ecological principles shape and drive the long-term dynamics and evolution of microbial ecosystems (Zampieri and Sauer, 2016). However, for the facultative parasite, the short-term nutritional history may alter its further pathogenic and saprophytic activity, or as for *P. setifera* also endophytic activity, manifested by shifts in their catabolic and genetic features. In this context, it was hypothesized that *P. setifera* compost isolate, precultured on three different wastes [oak sawdust, beet pulp (BP) and wheat bran (WB)] would reveal different metabolic properties and genetic fingerprints. These results might be useful in the prediction of possible pathogenesis/degradation of different plants. Therefore, the aim of this study was to evaluate the influence of waste on *P. setifera* metabolic and genetic profile.

## Materials and Methods

### Waste Material

The three following waste materials were considered: oak sawdust (SD), dried BP and durum WB. The wastes were powdered using a ball mill Retsch MM 400, 30 Hz for 15 min. The chemical analysis of wastes such as total solids (TS), volatile solids (VS), crude ash (CA), crude fat (CF), total nitrogen (N_tot_), crude protein (CP), non-fiber carbohydrates (NFC) and crude fiber fraction: neutral detergent fiber (NDF), acid detergent fiber (ADF), and acid detergent lignin (ADL) were conducted as described by Oleszek and Krzemińska (2016). Briefly, TS, VS, and CA were investigated with a muffle furnace after drying at 105 and 550°C, respectively. CF was determined by extraction with hexane. Total nitrogen was analyzed using the Kjeldahl method, and then N_NO3_ and N_NH4_ were subtracted, leaving the organic nitrogen value (N_org_). CP was calculated by multiplying N_org_ by a coefficient of 6.25. NDF, ADF, and ADL were evaluated with van Soest and Wine’s method (Van Soest, 1963). Based on NDF, ADF, and ADL results, the cellulose (CEL) and hemicellulose (HCEL) contents were calculated by subtracting ADF from NDF and ADL from ADF, respectively. NFC were estimated by using the following formula:

NFC = 100% - (CP + CF + NDF + CA)

where NFC is NFC (% TS), CP is crude protein (% TS), CF is crude fat (% TS), NDF is neutral detergent fiber (% TS), and CA is crude ash (% TS), where TS is TSs.

The phosphorus level (P) was determined calorimetrically and potassium (K) was estimated by flame photometry according to the Spurway method (Spurway and Lawton, 1949). N_org_, N_NO3_, N_NH4_, P, and K analyses were determined at District Chemical and Agricultural Station in Rzeszów. The results were obtained as mean values. All chemical analyses of tested waste were performed in triplicates.

### *Petriella setifera* Isolates

The five fungal strains G11/16, G14/16, G16/16, G17/16, G18/16 were selected from among the fungal collection of the Laboratory of Molecular and Environmental Microbiology, Institute of Agrophysics Polish Academy of Sciences (Lublin, Poland). These were isolated from industrial composts using a serial dilution method on Bengal Rose LAB-AGAR medium (BIOCORP, Poland) and identified as *P. setifera* using two approaches. These were based on the D2 domain of Large-Subunit ribosomal DNA (D2 LSU rDNA) and Internal Transcribed Spacer 1 rRNA (ITS1) sequencing (Thermo Fisher Scientific, United States). Nucleotide sequences of the strains were deposited in the National Centre for Biotechnology Information (NCBI) under the following accession numbers: KX639331, KX639334, KX639335, KX639336, KX639337, respectively, following D2 LSU rDNA sequencing, and: MG594608, MG594609, MG594610, MG594611, MG594612, following ITS1sequencing. The industrial compost consisted of sewage sludge from the treatment of wastewater, sawdust, biodegradable waste from gardens and from parks, soil, the extracts of medicinal plants, and lime sludge. The concentration of carbon, nitrogen and phosphorus in the compost were 17.9, 2.3, and 0.75%, respectively, and pH was 5.3.

### Waste Decomposition Using MT2 Plates^®^

*Petriella setifera* isolates were evaluated in four replicates against waste substrate decomposing abilities based on the growth intensity on powdered waste-based substrate, such as oak sawdust, BP, WB (prepared as described above), using MT2 plates^®^ manufactured by Biolog^®^. To prepare an inoculum, each isolate was cultivated on Potato Dextrose Agar medium (PDA) (Oxoid Ltd., England) with a 3% addition of oak sawdust (SDM), beet pulp (BPM) or, wheat bran (WBM), and control medium (CLM) without any additives, at 27°C in the dark, for 25 days including 7 days with white light exposure for spore formation. 100 μl of the mycelium water suspension was added to wells on the MT2 plate, where previously 50 μl of 1% SD, BP or WB water solution was placed, following the modified procedures of Kadali et al. (2012), Taha et al. (2015) and Frąc et al. (2016), in four replicates. The inoculated microplates were incubated at 27°C for 10 days. The optical density (OD) at 750 nm was determined every 24 h using a microplate reader.

### The Catabolic Profile of *Petriella* Fungi Using FF Plates^®^

The catabolic profiles of *P. setifera* isolates were generated from FF plates^®^ based on the growth intensity of the organism on 95 low-molecular-weight carbon sources. The inoculation procedure was based on the FF plate^®^ method according to the manufacturer’s protocol modified by Frąc et al. (2012). The inoculation procedure was performed as for the MT2 plate analyses. After the homogenization of the mycelium suspension in inoculating fluid (FF-IF, Biolog^®^) the transmittance was adjusted to 75% using a turbidimeter (Biolog^®^). 100 μl of the mycelium suspension was added to each well and microplates were incubated at 27°C for 10 days. The OD at 750 nm was determined using a microplate reader every 24 h, in four replicates. Functional diversity was determined by the number of different substrates utilized by the individual isolates and expressed as substrate Richness (R), and Average Well-Density Development (AWDD) index calculated as following Average Well-Colour Development (AWCD) (Frąc et al., 2012), based on OD readings.

### Genetic Diversity Based on AFLP and cAFLP

From each of the five strains cultured on SD, BP, and WB, 200 mg of fungal mycelium was taken and sterilely transferred into 2 ml tubes containing 250 mg of glass beads of 1.45 mm diameter. Then, 500 mg of glass beads of 3.15 mm diameter and they were homogenized with FastPrep-24 homogenizer (MP Bio, United States) for 20 s at 4 m/s. The DNA was extracted in accordance to EURx GeneMATRIX Plant and Fungi DNA Purification Kit (EURx, Poland) protocol. The quantity and purity of extracted DNA were evaluated with NanoDrop-2000 Spectrophotometer (Thermo Scientific, United States).

The AFLP reactions were performed with the use of *Pst*I and *Mse*I restriction enzymes. The results of the analysis were visualized by capillary electrophoresis with an Applied Biosystems 3130 Genetic Analyser (Applied Biosystems, United States). The sequences of adapters (5′–3′) and primers used in this study are denoted: *Mse*I_AF GAC GAT GAG TCC TGA G; *Mse*I_AR TAC TCA GGA CTC AT; *Pst*I_AF CTC GTA GAC TGC GTA CAT GCA; *Pst*I_AR TGT ACG CAG TCT AC; 6-FAM-*Pst*I+ACA ^∗^FAM- GAC TGC GTA CAT GCA GAC A; *Mse*I+CA GAT GAG TCC TGA GTA ACA. The AFLP reactions were performed in three biological replications for each isolate. The double-stranded *Pst*I and *Mse*I oligonucleotide adapters were formed in a final volume of 2 μl by incubating 0.5 μl of 10 μM *Pst*I_AF, 0.5 μl of 10 μM *Pst*I_AR, 0.5 μl of 100 μM *Mse*I_AF and 0.5 μl of 100 μM *Mse*I_AR adapters at 95°C for 5 min, followed by 15 min at room temperature. Successively, the restriction-ligation (RL) reaction was performed. The genomic DNA (500 ng) was digested with 5 U of the *Pst*I restriction enzyme (EURx, Poland) and 5 U of the *Mse*I restriction enzyme (New England Biolabs, United States). The RL solution was composed of 1 U of T4 DNA Ligase (EURx, Poland), 2 μl of double-stranded adapters, 50 mM Tris-HCl, 10 mM MgCl_2_, 10 mM DTT, 1 mM ATP and 25 μg/ml of BSA in a final volume of 20 μl.

The RL reaction was carried out for 1 h at 37°C. At the end of this reaction, each RL reaction mixture was diluted with an addition of 80 μl of sterile water and 1 μl of this solution was used as a template in the selective amplification reaction. The selective PCR amplification reaction was performed in a final volume of 5 μl which consisted of 2.5 μl of 2X Taq PCR Reaction Master Mix (EURx, Poland), 1 μl of diluted RL solution, 0.25 μl of 10 μM 6-FAM-*Pst*I+ACA primer (Genomed, Poland) and 0.25 μL of 10 μM *Mse*I+CA primer (Genomed, Poland). The reaction was performed in a Verti Fast thermal cycler (Applied Biosystems, United States) under the following conditions: 72°C for 120 s, followed by 7 cycles of 94°C for 15 s, 63°C with a touchdown of -1°C by cycle for 30 s, 72°C for 45 s, followed by 33 cycles of 94°C for 45 s, 56°C for 30 s, 72°C for 45 s and followed by a final step at 72°C for 60 s. At the end of this step, the exonuclease I – alkaline phosphatase purification step was performed. Hence, 2 μl of Exo-BAP Mix (EURx, Poland) was added to each reaction tube. The samples were incubated at 37°C for 15 min and then at 80°C for another 15 min. Next, 28 μl of sterile water was added into each PCR-product and 0.5 μl of this solution was combined with 0.25 μl of GS-600 LIZ Standard (Applied Biosystems, United States) and 9.25 μl of HiDi formamide (Applied Biosystems, United States). This mixture was incubated for 150 s at 95°C and cooled down in ice for 5 min. The amplicons were separated by capillary electrophoresis with an Applied Biosystems 3130 Genetic Analyser (Applied Biosystems, United States), in a 50 cm capillary array filled with NanoPOP-7 Polymer (McLAB, United States). The fragments were compared to the standard and visualized in the form of an electropherogram using a GeneMapper^®^ version 4.0 software (Applied Biosystems, United States).

The cAFLP was performed on complementary DNA (cDNA). Therefore, total RNA was extracted from each of the five strains cultured on SD, BP, and WB. The RNA was extracted by a MagMAX^TM^-96 Total RNA Isolation Kit (Thermo Fischer) with a modified homogenization step. 100 mg of mycelium was suspended in 1 ml of nuclease-free water. Then the samples were mixed for 3 min and 165 μl of suspension was transferred on bead tubes (Thermo Fischer) containing 235 μl of Lyse F buffer (EURx). Then homogenization with a FastPrep-24 instrument (MP Bio) at 6.5 m/s with two cycles of 1 min each separated by 2 min of rest was performed. After that, samples were centrifuged at 16000 rcf for 210 s and 200 μl of lysate was transferred to a new tube. To clarify the lysate we centrifuged samples at 16000 rcf for 360 s.

After this step, the MagMAX^TM^-96 Total RNA Isolation Kit protocol was performed. A reverse transcription was performed using a High-Capacity cDNA Reverse Transcription Kit (Thermo Fischer) with the addition of 10 μM of anchored Oligo(dT)_20_ Primer. At the end of this step, the purification of exonuclease I – bacterial alkaline phosphatase was performed by the addition of 2 μl of Exo-BAP Mix (EURx, Poland) to 5 μl of each sample. The samples were incubated at 37°C for 15 min and then at 80°C for another 15 min.

The double-stranded *Pst*I and *Mse*I oligonucleotide adapters were formed as described for AFLP. The RL reaction was performed. The cDNA was digested with 5 U of the *Pst*I restriction enzyme (EURx, Poland) and 5 U of the *Mse*I restriction enzyme (New England Biolabs, United States). The RL solution was composed of 1 U of T4 DNA Ligase (EURx, Poland), 2 μl of double-stranded adapters, 50 mM Tris-HCl, 10 mM MgCl_2_, 10 mM DTT, 1 mM ATP, and 25 μg/ml of BSA in a final volume of 20 μl.

The RL reaction was carried out for 1 h at 37°C. At the end of this reaction, each RL reaction was diluted by the addition of 80 μl of nuclease-free water and 1 μl of this solution was used as a template in the preamplification reaction. The preamplification reaction was performed in a final volume of 5 μl, which consisted of 2.5 μl of 2X Taq PCR Reaction Master Mix (EURx, Poland), 1 μl of diluted RL solution, 0.25 μl of 10 μM PstI+A primer (Genomed, Poland), and 0.25 μl of 10 μM MseI+C primer (Genomed, Poland). The reaction was performed in a Veriti Fast thermal cycler (Applied Biosystems, United States) under the following conditions: 72°C for 120 s followed by 35 cycles of 95°C for 15 s, 50°C for 30 s, 72°C for 45 s, and followed by a final step at 60°C for 30 s. After this, each reaction mixture was diluted by the addition of 45 μl of nuclease-free water and 1 μl of this solution was used as a template in the selective amplification reaction.

The selective amplification reaction was performed in a final volume of 5 μl, which consisted of 2.5 μl of 2X Taq PCR Reaction Master Mix (EURx, Poland), 1 μl of diluted preamplification solution, 0.25 μl of 10 μM 6-FAM-PstI+ACA primer (Genomed, Poland), and 0.25 μL of 10 μM MseI+CA primer (Genomed, Poland). The reaction was performed in a Veriti Fast thermal cycler (Applied Biosystems, United States) under the following conditions: 72°C for 120 s followed by 7 cycles of 94°C for 15 s, 63°C with a touchdown of -1°C per cycle for 30 s, 72°C for 45 s followed by 33 cycles of 94°C for 45 s, 56°C for 30 s, 72°C for 45 s, and followed by a final step at 72°C for 60 s. At the end of this step, purification by Exo-BAP Mix (EURx, Poland) was performed. In the next step, 28 μl of sterile water was added into each selective amplification product and 0.5 μl of this solution was combined with 0.25 μl of GS-600 LIZ Standard (Applied Biosystems, United States) and 9.25 μl of HiDi formamide (Applied Biosystems, United States). This mixture was incubated for 150 s at 95°C and cooled down in an ice bath for 5 min. The transcriptome-derived fragments were separated by capillary electrophoresis as described for AFLP.

### Statistical Analyses

Analysis of variance (ANOVA) was used to determine the differences in functional diversity indices and lignocellulose substrate utilization. *Post hoc* analyses were performed using a Tukey test (HSD). The data were presented as 95% confidence intervals. Statistical significance was established at *p* < 0.05. Pearson correlation coefficients (*p* < 0.05) were calculated between each characteristic and OD of *P. setifera* measured in the MT2 plate, within all the waste taken into consideration, values in bold indicate a strong correlation. Additionally, the cluster analysis for waste utilization (MT2 microplates), catabolic profiles (FF microplates) and genetic profiles (AFLP and cAFLP) on tested *P. setifera* isolates was used to detect groups in the data set through the calculation of Euclidian distance using the Ward method approach, which was calculated based on the average of all readings. The obtained results were depicted using dendrograms calculated using the Ward method cluster analysis with Sneath’s dissimilarity criteria (single bindings agglomeration and Euclidean distance measure). All statistical analyses, described above were performed using Statistica software (version 10.0).

## Results and Discussion

Intraspecific variation is a common feature of fungi which has been widely investigated on both a metabolic (Knapp and Kovács, 2016; Wang et al., 2016) and genetic level (Fedorova et al., 2009; Corradi and Brachmann, 2016). In this study the influence of three different materials (oak sawdust, dried BP, durum WB) on *P. setifera* catabolic and genetic properties were tested. The chemical characteristics of these materials are presented in **Table 1**. All wastes were represented by similar TS, VS, and CA content. WB encompassed the highest content of CF (3.77% TS), CP (17.94% TS), and NFC (28.63% TS), compared with oak sawdust and BP. WB also contained a relatively high amount of phosphorus (P) (6151 ppm), potassium (K) (10793 ppm), and nitrogen (N): 2.87 ppm, 47.11 ppm and 606 ppm of N_org_, N_NO3_, and N_NH4_, respectively. Sawdust was in turn distinguished by a high content of all fractions of fiber: NDF (85.47% TS), ADF (55.17% TS), particularly ADL 14.35% TS) and a very low content of CA and macroelements (N, P, K), as well as CP. BP was characterized by a higher content of hemicellulose (HCEL), compared to other wastes and the lowest content of ADL. The results of chemical analyses were in accordance with other studies on similar materials (Mikiashvili et al., 2011; Stevenson et al., 2012).

**Table 1:** Chemical properties of tested wastes.

Characteristics	Wheat bran	Oak sawdust	Beet pulp	Correlation coefficient
TS (%)	91.61 ± 0.11	94.11 ± 0.13	92.53 ± 0.09	0.22
VS (% TS)	96.26 ± 0.11	99.44 ± 0.05	95.99 ± 0.07	0.22
CA (% TS)	3.74 ± 0.12	0.56 ± 0.04	4.01 ± 0.08	0.48
N_org_ (% TS)	2.87	0.12	1.46	**0.79**
N_NO3_ (ppm)	47.11	15.50	27.80	**0.59**
N_NH4_ (ppm)	606.67	84.62	528.00	**0.73**
P (ppm)	6151.11	469.23	808	**0.91**
K (ppm)	10793.33	426.92	5808	**0.78**
CP (% TS)	17.94	0.75	13.51	**0.80**
CF (% TS)	3.77 ± 0.50	1.01 ± 0.08	0.31 ± 0.05	-0.17
NFC (% TS)	28.63 ± 1.77	12.21 ± 3.71	23.11 ± 0.05	**0.567**
NDF (% TS)	45.78 ± 0.92	85.47 ± 3.04	59.06 ± 0.04	-0.12
ADF (% TS)	12.06 ± 1.68	55.17 ± 1.23	23.43 ± 1.49	-0.35
ADL (% TS)	2.00 ± 0.15	14.35 ± 2.09	1.36 ± 0.04	-0.29
CEL (% TS)	11.03 ± 0.04	40.82 ± 2.09	22.07 ± 0.14	-0.35
HCEL (% TS)	32.89 ± 1.18	30.30 ± 2.23	35.63 ± 0.18	0.22
(CEL+HCEL)/ADL	21.96	4.95	42.43	

*TS, total solids; VS, volatile solids; CA, crude ash; CF, crude fat; N_*org*_, organic nitrogen; N_*NO3*_, nitrate nitrogen; N_*NH4*_, ammonium nitrogen; P, phosphorus; K, potassium; NFC, non-fiber carbohydrates; NDF, neutral detergent fiber; ADF, acid detergent fiber; ADL, acid detergent lignin; CEL, cellulose; HCEL, hemicellulose; NFC, non-fiber carbohydrates; *n* = 3. Pearson correlation coefficients (*p* < 0.05) were calculated between each characteristic and optical density comprising the ability of *Petriella setifera* to decomposition of waste measured in MT2 plate, within all the waste taken under consideration. The bolded values indicate significant correlation coefficients with *p* < 0.05.*

Among the waste tested, the highest ratio of hemicellulose to lignin [(CEL+HCEL)/ADL] (42.43) for BP was evidenced, whereas in WB the ratio reached 21.96. The lowest ratio (4.95) was observed for sawdust. As mentioned, e.g., by Lyson and Sobolewska (2015) the high hemicellulose to lignin ratio means that this waste was regarded to be the most susceptible to biological decomposition, and therefore difficult, if the ratio is low. This is only partially consistent with our study, because for *Petriella*, a rather weak negative correlation was noted with lignocellulose components, such as: NDF, ADF hemicellulose and lignin content (ADL), lignin (ADL), cellulose content (CEL) as well as CF (**Table 1**). At the same time, a strongly positive correlation with N, P, K, protein (CP) and simple sugars soluble in water, namely NFC was recorded. This means that *P. setifera* compost isolates are more capable of decomposing materials rich in protein, N, P, K and easily accessible sugars, than lignocellulose. Furthermore, improved decomposition of WB compared to BP was revealed, in spite of an almost twofold lower hemicellulose-to-lignin ratio. Therefore, more important for the decomposition ability of *P. setifera* is the accessibility of N, P, K and protein in the material, rather than the limiting hemicellulose to lignin ratio.

**FIGURE 1:**
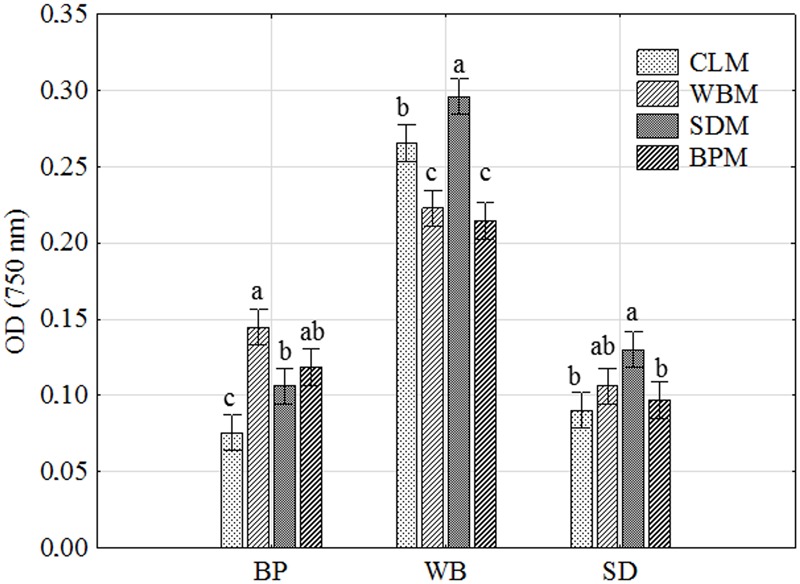
*Petriella setifera* decomposition ability of waste-based substrate located in the MT2 plate^®^ as influenced by preculturing. SD, oak sawdust; BP, beet pulp; WB, wheat bran; SDM, oak sawdust medium; BPM, beet pulp medium; WBM, wheat bran medium; CLM, control medium; OD, optical density. The vertical bars indicate the confidence intervals at 0.95. Different letters above the bars indicate errors in the significance of differences (α = 0.05) between the mean values of OD [Tukey’s test (*p* < 0.05)], *n* = 4.

As presented in **Figure 1**, *P. setifera* was the most effective at decomposing such a waste substrate located on MT2 plates^®^ as WB. BP and sawdust were generally significantly less rapidly decomposed. Notwithstanding it occurred that preculturing, nutritional condition with a high lignocellulose content significantly contributed to the improved ability of *P. setifera* to utilize substrates in MT2. The variability in that waste utilization was revealed. BP was preferably decomposed to a significant extent if the fungus is precultured with any waste-based medium [bran medium (WBM)], sawdust medium (SDM) or beet pulp medium (BPM), compared to the control (CLM). As far as WB is concerned, its utilization was significantly enhanced by preculturing on SDM, and significantly decreased if precultured on WBM and BPM. When it comes to sawdust, it was noticed that the preculturing of *P. setifera* on SDM significantly improved sawdust utilization, whereas just slightly if precultured on WBM. As tough nutritional condition (high lignocellulose content) as encountered while preculturing *P. setifera* on SDM, triggered genetic and metabolic changes at the cellular level clearly and were subsequently persistent regardless of the changed material. It is supposed that nutritional short-term history may result in the facilitation of the switching ability of *P. setifera* to follow a pathogen/saprophyte or endophyte mode of action.

**FIGURE 2:**
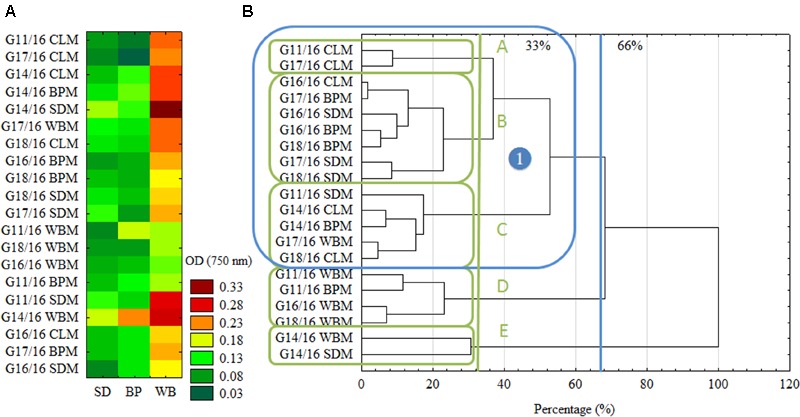
Intraspecific metabolic variability of *P. setifera* isolates as influenced by preculturing. **(A)** The cluster analysis depending on the waste-based substrate decomposition in MT2 plate^®^, **(B)** clustering according to the stringent Sneath’s criterion (33%) and less restrictive criterion (66%). For explanations: please see **Figure 1**.

What is more, the intraspecific differences among *P. setifera* isolates were also described for waste utilization in MT2 Plate^®^ (**Figure 2A**). Most intensive catabolic properties for WB utilization were noted for strains G14/16 and G11/16 precultured on SDM. On the other hand, G18/16 and G16/18 strains precultured on WBM were the least active for this waste. Among strains tested against waste utilization in the MT2 plate^®^, there were five (A–E) groups revealed if the restrictive Ward’s criterion (33%) was taken into consideration (**Figure 2B**). Groups A–C comprised group (1), standing out by comprising strains G16/16, G17/16 and G18/16. In group A, there were strains precultured on CLM clustered, in B and C mostly on SDM and BPM, and D on WBM. E and C groups were characteristic in so far as both strains of G14/16 were located. This suggests one notable intraspecific difference in metabolic properties of *P. setifera* as influenced by culture media composition.

**FIGURE 3:**
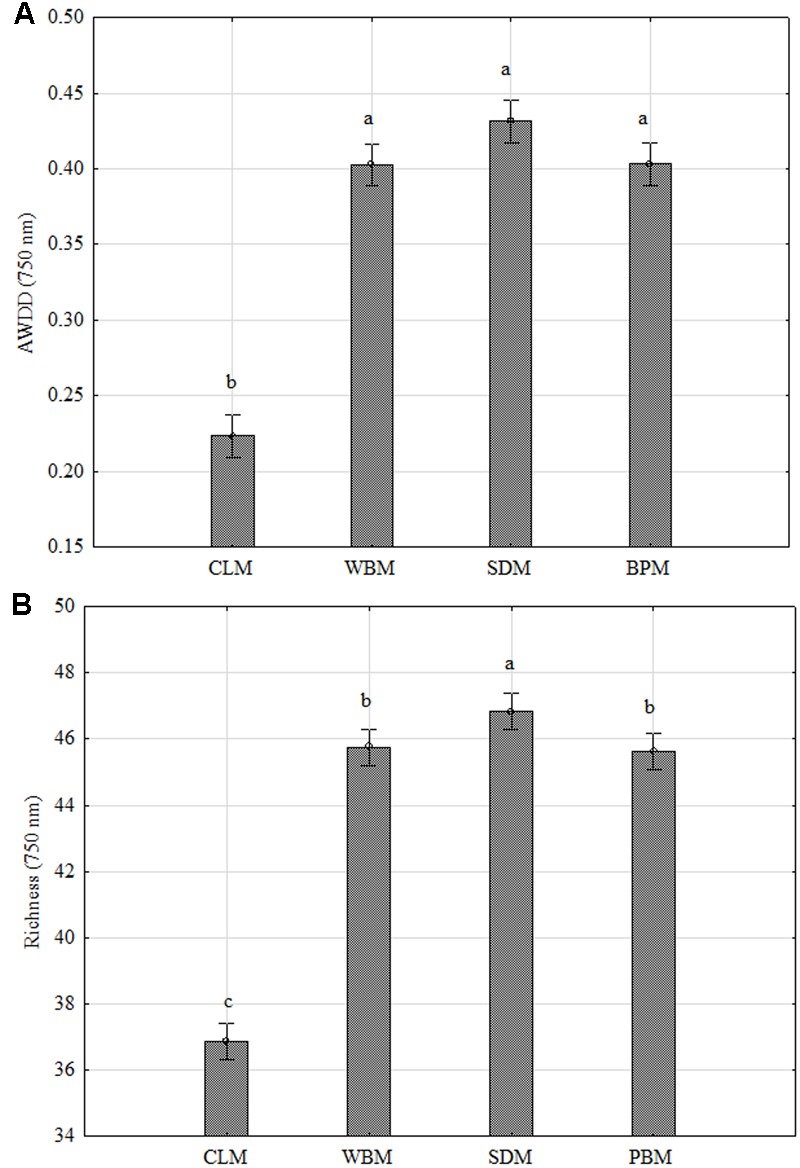
FF plate^®^ biodiversity indices based on carbon compounds utilization ability of *Petriella setifera* as influenced by preculturing. **(A)** Average Well-Density Development index (AWDD), **(B)** substrate Richness (R). Explanations: SDM - oak sawdust medium, BPM - beet pulp medium, WBM - wheat bran medium, CLM - control medium. The vertical bars indicate the confidence intervals at 0.95. Different letters above the bars indicate errors in the significance of differences (α = 0.05) between the mean values of OD [Tukey’s test (*p* < 0.05)], *n* = 3.

**FIGURE 4:**
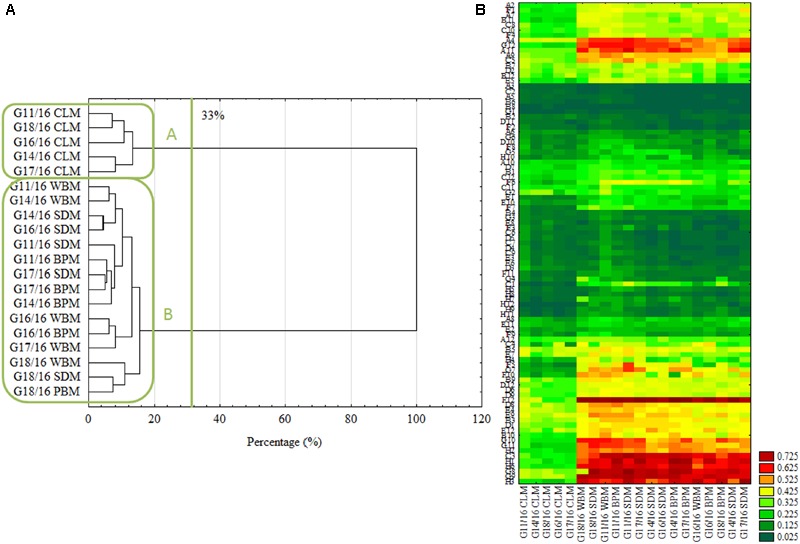
Intraspecific metabolic diversity of *P. setifera* isolates as influenced by preculturing. **(A)** Clustering according to the stringent Sneath’s criterion (33%) and less restrictive criterion (66%), **(B)** the cluster analysis depending on carbon-compound utilization located in the FF plate^®^. For explanations: please see **Figure 1**. Carbon compounds decoding according to (Frąc, 2012).

In **Figure 3** biodiversity indices are presented: AWDD and Richness (R) calculated based on FF plates^®^. It shows the significant dependency of *P. setifera* abilities to catabolize C-compounds in FF plates^®^, on preculturing on media with all the tested waste additives (WBM, SDM, BPM), twice compared to the control as far as AWDD. Among tested media SDM significantly influenced metabolism of *P. setifera* to increase the number of utilized substrates, which was evidenced by a 20% upswing in Richness. The result for C-compounds are consistent with the more complex substrates as tested in MT2. However, there was less diversity among strains found based on the FF approach, compared to MT2, which was shown in **Figure 4**.

In accordance with the previous report (Eisen et al., 1998) the cluster analysis revealed clear patterns of microbial properties. Similar research on FF plates^®^ showing metabolic differences of closely related fungi were recently performed by, e.g., (Janusz et al., 2015; Jaber et al., 2017; Pinzari et al., 2017). Regardless of the restriction criterion in cluster analysis, *P. setifera* isolates revealed dichotomy clustering in A and B groups (**Figure 4A**). There were *P. setifera* strains that were precultured on CLM included in the A group. Whereas all the other strains of initially precultured variants comprised a separate group B. The catabolic activity of *P. setifera* precultured on waste was highly strengthened toward more effective utilization of some particular C-compounds located on the FF plate^®^ (**Figure 4B**). These were particularly: *N*-Acetyl-D-Glucosamine (A4), L-Arabinose (A9), Arbutin (A11), belonging to Carbohydrates; D-Glucuronic Acid (C3) and Quinic Acid (F12), which contain the carboxylic and ketonic acid groups; polymer Glycogen (C5); L-Alanine (G8), L-Alanyl-Glycine (G9), L-Asparagine (G10), L-Aspartic Acid (G11), L-Glutaminic Acid (G12), Glycyl-L-Glutamic Acid (H1), L-Ornithine (H2), L-Phenylalanine (H3), L-Serine (H6) that belong to the amino acid class. At a slightly lower level the utilization of γ-Amino-Butyric Acid (F1), D-Mannose (D2), D-Xylose (E12), L-Rhamnose (D12) was also increased. These results were in compliance with the properties of soft-rot fungi (Schwarze, 2007; Mathieu et al., 2013). It was found that all of the isolates could degrade at a high level the substances that may be produced during the hemicellulose’s and cellulose’s degradation. Furthermore, it was revealed that all of the analyzed isolates degraded Quinic Acid, which is a monomer of lignin degraded by brown-rot fungi at a high rate (Albrecht et al., 2010). Both modes of action are characteristic for fungal pathogens and/or saprophytes. Verma et al. (2007) described that plant fungal pathogens typically secrete a number of plant cell-wall-degrading enzymes (cellulases, glucanases, polygalacturonases, xylanases), and may persist on dead plants as a saprophyte. However, fungal endophytes being in a mutual relationship with a plant must lose their ability to produce hydrolytic enzymes and already use simple compounds provided by the plant. Many of the C-compounds effectively utilized by *Petriella* are plant cell metabolites (Kot et al., 2015), that *Petriella* uses as being an endophyte. What is more, the growth of all tested *P. setifera* isolates was inhibited by amines and amides, especially by *N*-Acetyl-D-Mannosamine and Glucuronamide (**Figure 4B**). In conclusion, the FF Plates^®^ results have also demonstrated slight intraspecific variability of the analyzed *P. setifera* strains to utilize particular C-compounds. *P. setifera* precultured on all waste-based media also revealed a wider diversity of C-compounds utilization, compared to the control. These results are consistent with MT2 findings and indicate that the nutritional history imposes actual metabolic activity which may trigger *P. setifera* mode of behavior. This may pose a threat to the corps especially if they are fertilized with compost inhabited by *P. setifera*. Plant pathogens in compost constitute a serious problem (Bollen and Volker, 1996). Reliable analyses are needed for the evaluation of infestation of the finished compost product, as a soil conditioner.

**FIGURE 5:**
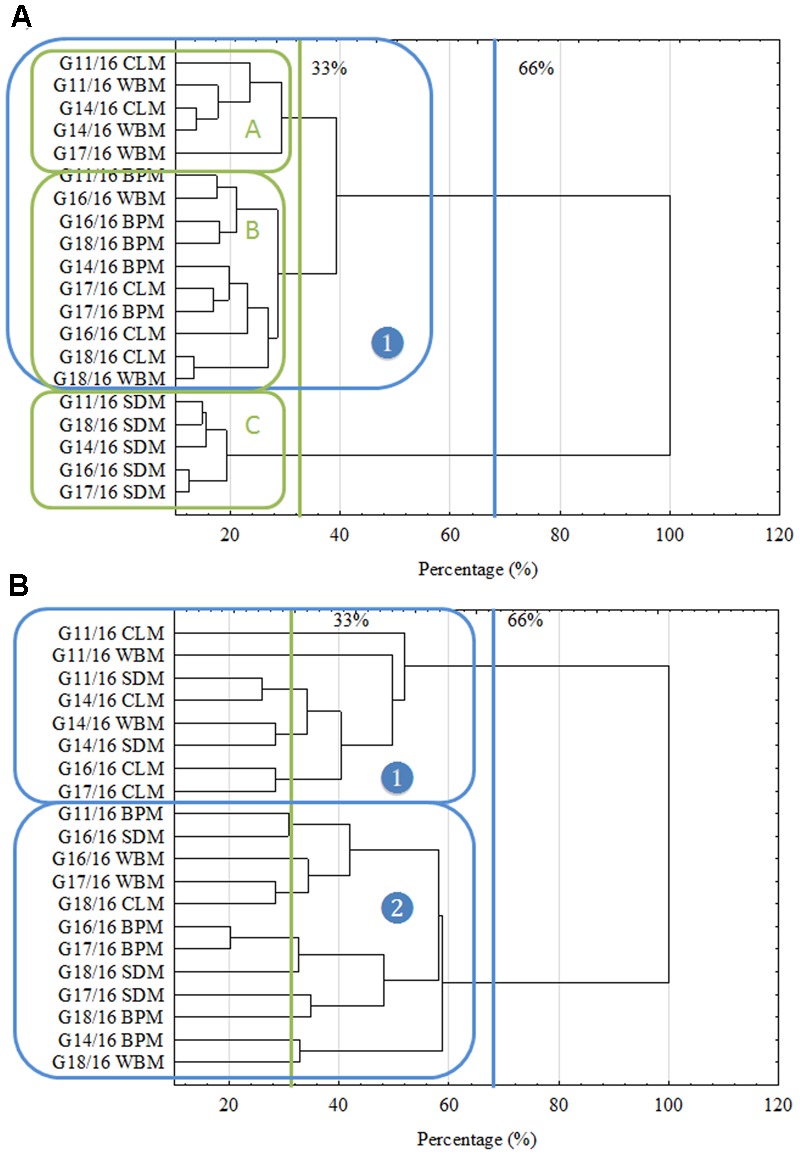
Intraspecific genetic fingerprinting of *P. setifera* isolates as influenced by preculturing. **(A)** Genome-derived fragments clustering, **(B)** transcriptome-derived fragments clustering based on Amplified Fragment-Length Polymorphism analysis. For explanations: please see **Figure 1**.

Trying to link the metabolic differences described above with genetic shifts, Amplified Fragment-Length Polymorphism (AFLP) was performed. Also cAFLP genotyping was provided, showing shifts in transcript-derived fragments (**Figure 5B**). These approaches were previously presented for fungi, e.g., by Al-Hatmi et al. (2016) and Xiao et al. (2016). In our findings there were three different groups noted for AFLP fingerprinting based on the 33% criterion (**Figure 5A**). Groups A and B included isolates cultured on all the wastes, besides SDM, which formed an outlying cluster C. This guarantees a clarification of the findings of the metabolic properties of *P. setifera*. All of the changes which may be observed at a metabolic level are determined by many modifications at a genetic level. This might be accentuating the expression or including the expression of other genes that code enzymes responsible for decomposition, or/and launching new metabolic pathways. This could also be the result of post-translational modifications of enzyme proteins (Brown, 2006) or epigenetic phenomena defined by reversible heritable changes in gene expression in the absence of changes in DNA sequence. These include, among others, DNA methylation, position effects, RNA silencing systems, and centromere location. Fungi share silencing systems, for instance RNA interference (iRNA) and DNA methylation (Smith et al., 2012). Similarly fingerprinting for isolates cultured on SDM resulted in specific DNA methylation since the *Pst*I restriction enzyme used is sensitive to cytosine methylation, predominately present at CpG or CpNpG sites. To be specific, *Pst*I is highly sensitive to the cytosine status in CpNpG sites because its recognition site involves two CpNpG trinucleotides (Cui et al., 2013). This may also be the effect of point mutations occurring on recognition sites: CTG CAG and TTAT for *Pst*I and *Mse*I, respectively (Montiel et al., 2006; Jiang et al., 2013). However, as was revealed by the cAFLP approach, other more complex scenarios were involved, since with transcriptome level modification no groupings were noted for particular isolates cultured on different wastes.

The influence of preculturing of *P. setifera* on selected waste type on metabolic and genetic properties was evidenced. Isolates were found to be better able to decompose waste materials such as WB and BP, rich in protein, N, P, K and easily accessible sugars compared to oak sawdust, rich in lignocellulose. Sawdust clearly triggered changes in the metabolic and genetic properties of *P. setifera*. However, intraspecific differences among *P. setifera* isolates were noted. Especially, the contribution to improve its ability to utilize waste substrates in the MT2 plate^®^ and the two times increase in the ability to catabolize carbon compounds located in FF plates^®^ was noted. Vivid metabolic properties following the preculturing of *Petriella* isolates on sawdust were in accordance with differing genotype profiles but not the transcriptome. Based on the study we may also conclude that amines and amides inhibited the growth of *P. setifera* isolates. Therefore, such compounds may be tested as potential agents in plant protection against this pathogen.

## Author Contributions

Conceived and designed the experiments: KO, JP, GP, AS, MO, and MF. Performed the experiments: KO, JP, GP, and AS. Analyzed the data: KO, MF, JP, and MO. Contributed reagents/materials/analysis tools: KO, JP, GP, AS, MO, and MF. Wrote the paper: KO and MF. Improvement and acceptance of the last version of manuscript: KO, JP, GP, AS, MO, and MF.

## Conflict of Interest Statement

The authors declare that the research was conducted in the absence of any commercial or financial relationships that could be construed as a potential conflict of interest.
